# Protective Effect of *Opuntia dillenii* (Ker Gawl.) Haw. Seed Oil on Gentamicin-Induced Nephrotoxicity: A Biochemical and Histological Analysis

**DOI:** 10.1155/2021/2173012

**Published:** 2021-08-31

**Authors:** Mohamed Bouhrim, Noureddine Bencheikh, Hamada Imtara, Nour Elhouda Daoudi, Hamza Mechchate, Hayat Ouassou, Loubna Kharchoufa, Mostafa Elachouri, Hassane Mekhfi, Abderrahim Ziyyat, Abdelkhaleq Legssyer, Mohammed Aziz, Mohamed Bnouham

**Affiliations:** ^1^Laboratory of Bioresources, Biotechnology, Ethnopharmacology and Health, Faculty of Sciences, Mohammed First University, Oujda, Morocco; ^2^Faculty of Arts and Sciences, Arab American University Palestine, P. O. Box 240, Jenin, State of Palestine; ^3^Laboratory of Biotechnology, Environment, Agrifood, and Health, University of Sidi Mohamed Ben Abdellah, Faculty of Sciences Dhar el Mahraz, Fez B.P. 1796, Morocco

## Abstract

*Opuntia dillenii* is a medicinal plant with frequent usage in folk medicine to treat many illnesses. The present study aims to investigate the protective effect of *Opuntia dillenii* seed oil against gentamicin-induced nephrotoxicity in rats. The animals (rats) were randomly divided into three groups (i) the normal control group treated only with distilled water (10 mL/kg), (ii) the gentamicin group treated with distilled water (10 mL/kg) and received an intraperitoneal injection of gentamicin (80 mg/kg), and (iii) the group treated with the *Opuntia dillenii* seed oil (2 mL/kg) and also received an intraperitoneal injection of gentamicin (80 mg/kg). The rats received their following treatments for 14 consecutive days orally. Serum urea, creatinine, gamma-glutamyl transferase, albumin, and electrolyte levels were quantified as the markers of acute renal and liver failure. Besides, the kidney and liver relative weight, kidney malondialdehydes, and kidney histological analysis were determined. The results have shown that daily pretreatment with *Opuntia dillenii* seed oil (2 mL/kg) prevented severe alterations of biochemical parameters and disruptions of kidney tissue structures. In addition, the results of the present study showed for the first time that *Opuntia dillenii* seed oil reduced renal toxicity in gentamicin-induced nephrotoxicity in rats. Therefore, *Opuntia dillenii* seed oil may represent a new therapeutic avenue to preserve and protect renal function in gentamicin-treated patients.

## 1. Introduction

Kidneys are considered one of the major clearance organs in the human body. It has a key role in xenobiotic detoxification [1, 2]. However, many factors and diseases can be associated with or lead to renal impairment, one of them is the formation of reactive oxygen species (ROS) [3]. Gentamicin belongs to the antibiotic aminoglycoside group, and it is used against life-threatening Gram-negative bacterial infections [4]. A major complication associated with gentamicin treatment is nephrotoxicity [5–7]. It has been demonstrated that the proximal tubule cells of the renal cortex have a high ability to concentrate gentamicin several-fold more than plasma levels [8]. The mechanism of nephrotoxicity by gentamicin is unclear. However, the effect of this drug might be related to the interactions with cell membrane, mitochondria, lysosomes, and microsomes [8]. Many researchers have linked those mechanisms to an increased lipid peroxidation mediated by the generation of reactive oxygen species, causing a deficiency in the kidney's intrinsic antioxidant enzymes [6–9]. Several experimental studies and improvement strategies have been conducted in an attempt to prevent and protect against gentamicin-mediated renal damage in vivo and in vitro [9–11]. Natural products, including *Opuntia dillenii* seeds oil (ODSO), are an important source of secondary metabolites (flavonoid and phenolic compounds) that may play a significant role in the prevention and treatment of kidney diseases [12, 13]. ODSO is an oil that is characterized by a great level of unsaturation fatty acids, wherein linoleic acid is the most common fatty acid, beta-sitosterol as the phytosterol marker, and *γ*-tocopherol as vitamin E [14]. Numerous studies explored the potential activities of this oil, including antioxidant [15], anti-inflammatory [16], hepatoprotective [17], and antidiabetic [18, 19]. The nephroprotective effects of ODSO against gentamicin are so far not well elucidated, and the present study aims to investigate it by assessing different parameters such as plasma biochemical parameters, oxidation tests, and renal tissue morphology in Wistar albino rats.

## 2. Materials and Methods

### 2.1. Chemicals

Alkaline phosphatase (ALP), gamma-glutamyl transpeptidase (GGT), urea, creatinine, albumin, and electrolyte kits were purchased from Biosystems, Spain. All other chemicals used were of analytical grade.

### 2.2. Collection of Plant Material

Fresh fruits of *Opuntia dillenii* used in this study were sampled from Essaouira, Morocco, in February 2019. The plant was authenticated by the expert botanist Mohammed Fennan, from the Scientific Institute of the University Mohammed 5, and the specimen was deposited in the Herbarium of the University Mohammed First, Oujda, Morocco, under the reference number HUMPOM 351.

### 2.3. Preparation of *Opuntia dillenii* Seeds Powder

The mature fruits of *Opuntia dillenii* were peeled; then, the seeds were isolated, washed with distilled water, dried in an oven at 37°C for three days, and then grounded using a blender until a fine and homogeneous powder was obtained and conserved at −20°C until use.

### 2.4. Oil Extraction

Grounded seed (100 g) was mixed with petroleum ether (500 mL) for 24 h under constant agitation at ambient temperature to get the oil content. The resulting extract was filtered and dried (rotary evaporator, 40°C). The oil was stored at 4°C.

### 2.5. Animals

Eighteen healthy adult Wistar rats ((200 ± 50) g, 10 weeks old) were employed in this research. The rats were provided from Animal House, Faculty of Sciences of Mohammed First University (Oujda, Morocco), which were housed in clean plastic cages at room temperature and in a humidity-controlled facility (22–26°C, with a 12 h light/dark cycle) with free access to water and food ad libitum. The animals were treated following the Guide for the Care and Use of Laboratory Animals of Research Council published by the US National Institutes of Health (NIH Publication No. 85-23, Revised in 1985). This study was approved by the Institutional Review Board of the Faculty of Sciences, Oujda, Morocco (02/20-LBBEH-04).

### 2.6. Determination of the Nephrotoxicity Dose Model

Two groups of Wistar rats were used to determine the dose of gentamicin for the induction of the experimental animal nephrotoxicity model. The normal control group (NCG) includes rats orally treated with vehicle (distilled water: 10 mL/kg) and the gentamicin group (GCG) includes rats who received distilled water and were injected intraperitoneally with 80 mg/kg of gentamicin 3 hours after the administration of distilled water. This treatment was carried out for 14 consecutive days. In this study, nephrotoxicity was induced in rats using daily intraperitoneal injections of GM, at a dose of (80 mg/kg; b.w) during all days of treatment. It is well known that the dose considered is commonly used to induce nephrotoxicity in experimental animals [20, 21]. After 24 h from the last injection, rats were deeply anesthetized and sacrificed, and then, their kidneys were isolated. Additionally, creatinine and urea plasmatic were measured.

### 2.7. Effect of ODSO on Gentamicin-Induced Nephrotoxicity in Rats

#### 2.7.1. Animal Groups

The animals were arbitrarily grouped into three equal groups each of six rats: the NCG group received distilled water 10 mL/kg orally and served as normal control. The GCG group was treated with distilled water 10 mL/kg orally and were injected intraperitoneally with 80 mg/kg of gentamicin. The *Opuntia* oil group (OOG) received gentamicin (80 mg/kg, i.p) and ODSO orally (2 mL/kg). The animals were daily injected with gentamicin three hours after the oil or distilled water administration for two weeks. The administration of the different treatments was by intragastric gavage using a stainless steel gavage needle with a pear-shaped tip to introduce them into the stomach. The body weights were recorded before and after 14 days of treatment. All animals were treated for 14 days.

#### 2.7.2. Blood Samples

Twelve hours after the last intraperitoneal injection of gentamicin, blood samples were collected from the treated rats after anesthesia. Thereafter, plasma was separated by centrifugation at 3000 rpm for 10 min at 4°C and after that stored at −20°C until analysis.

#### 2.7.3. Serum Biochemical Parameters Determination

Markers of renal impairment such as plasma creatinine, urea, alkaline phosphatase, gamma-glutamyl transpeptidase, and albumin were measured using the analyzer COBAS INTEGRA^®^ 400 plus.

#### 2.7.4. Determination of Malondialdehydes

The kidneys of the treated animals were used to prepare kidney homogenate (10% w/v) in normal saline (pH 7.0) and were conserved at −20°C. In this study, renal lipid peroxidation was measured according to the Buege and Aust method [22]. This method measures the level of TBARS production as described by Iqbal et al. [23]. After the preparation of the homogenate, a volume of 0.5 mL of kidney homogenate was mixed with 0.5 mL of 30% (w/v) trichloroacetic acid and subjected to centrifugation at 3500 rpm for 10 min at 4°C. 1 mL of thiobarbituric acid (0.67% w/v) was then added to 1 mL of the supernatant and kept in a boiling waterbath for 10 min. The mixture was placed in a cold ice bath to stop the reaction. A spectrophotometer was used to measure the mixture of assays at 535 nm, and the calculation was made using the next molar extinction coefficient:

(1) $$\begin{array}{c} 1.56\times 10^{5\;}\;{\text{m}}^{-1}{\text{cm}}^{-1}. \end{array}$$

The results were expressed in nanomoles of MDA produced per gram of tissue.

#### 2.7.5. Histological Analysis

In this study, the kidneys of the animals were prepared to examine the microscopic lesions. A buffered formalin solution (10%) was used to conserve the kidneys within 48 hours, and then, they were embedded in paraffin to perform a 4- 5 *μ*m thick tissue section cut using a rotating microtome. The mounted glass blades were kept on a heating plate (54°C) overnight. Finally, they were stained using hematoxylin and eosin (H&E) to visualize the different tissue components under a microscope.

### 2.8. Statistical Analysis

GraphPad Prism 5 for Windows was used to perform the statistical analysis. The data were expressed as means ± SEM. A statistically significant difference was estimated using a one-way analysis of variance (ANOVA), and the Bonferroni test was performed for multiple comparisons. Probability values (*p* < 0.05) were considered to be statistically significant.

## 3. Results

### 3.1. Determination of the Nephrotoxicity Dose Model

The treatment with gentamicin at 80 mg/kg/day for 14 days induced a significant renal impairment biomarker rise (uric acid, creatinine, and urea) compared to the normal control group (Table 1).

### 3.2. Effect of ODSO on Relative Right Kidney Weight and Bodyweight Gain Variations

The coadministration of ODSO and gentamicin to the rats did not induce an important change in the bodyweight gain and relative right kidney weight between all groups (Table 2).

### 3.3. Effect of Administration of ODSO on the Plasma Electrolyte Level

The effect of gentamicin and ODSO cotreatment on plasma electrolyte levels is presented in Table 3. In this study, the results showed that gentamicin administration did not alter the electrolyte levels significantly in the GCG and OOC groups compared to the NCG.

### 3.4. Effect of Administration of ODSO on the Plasma Urea and Creatinine Levels

**Figure 1:**
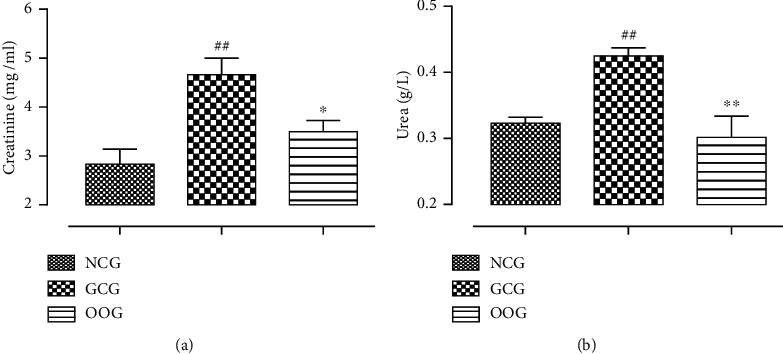
The amount of plasma creatinine (a) and urea (b) for all treated group animals. ^##^*P* < 0.01, compared to the NCG. ^*∗*^*P* < 0.05 and ^*∗∗*^*p* < 0.01, compared to the GCG.

The different groups of renal biomarkers levels in the plasma (urea and creatinine) are summarized in Figure 1. The intraperitoneal injection of gentamicin significantly (*p* < 0.01) increased the amount of urea and creatinine in the GCG animals, compared to the animals of the NCG group. However, coadministration of gentamicin with the ODSO has significantly counteracted the effect of gentamicin and decreased the plasma urea and creatinine concentration compared to the GCG group (*p* < 0.01 and *p* < 0.05, respectively).

### 3.5. Effect of ODSO Administration on Plasma GGT and ALP Levels

**Figure 2:**
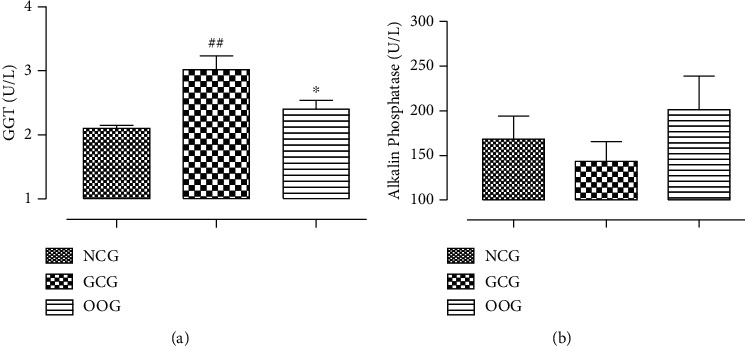
The amount of plasma GGT (a) and ALP (b) for all treated group animals. ^##^*P* < 0.01, compared to the NCG. ^*∗*^*P* < 0.05, compared to the GCG.

The effect of gentamicin administration alone or in association with ODSO on the liver biomarkers is shown in Figure 2. The results indicate that gentamicin intake significantly (*p* < 0.01) increased the GGT amount. However, the daily coadministration with ODSO has attenuated the effect of gentamicin with a significant (*p* < 0.05) decrease in the amount of GGT (Figure 1(a)). Contrariwise, the gentamicin intake with or without the ODSO does not induce a significant variation in the ALP level (Figure 1(b)).

### 3.6. Effect of Oral Administration of ODSO on the Plasma Albumin Level

**Figure 3:**
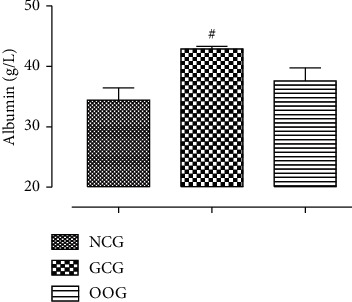
The concentration of plasma albumin in the treated animals. ^#^*P* < 0.05, compared to the NCG.

The concentration of plasma albumin was increased significantly (*p* < 0.05) in the animals treated with gentamicin. However, ODSO has counteracted the effect of gentamicin and decreased the concentration of albumin in plasma (Figure 3).

### 3.7. Effect of Oral Administration of ODSO on Lipid Peroxidation

**Figure 4:**
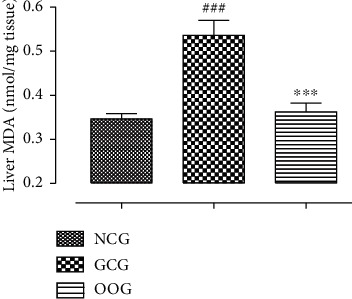
The amount of MDA produced in the kidney for all treated group animals. ^*∗*^*P* < 0.001, compared to the GCG. ^##^*P* < 0.001, compared to the NCG.

As shown in Figure 4, the effect of ODSO on lipid peroxidation of the kidneys was altered by the injection of gentamicin. The results of this study showed that daily injection of gentamicin caused a significant increase in the MDA amount (*p* < 0.001). However, the daily administration of ODSO decreased significantly (*p* < 0.001) the lipid peroxidation level in the kidney.

### 3.8. Protective Effect of ODSO against Renal Lesions

**Figure 5:**
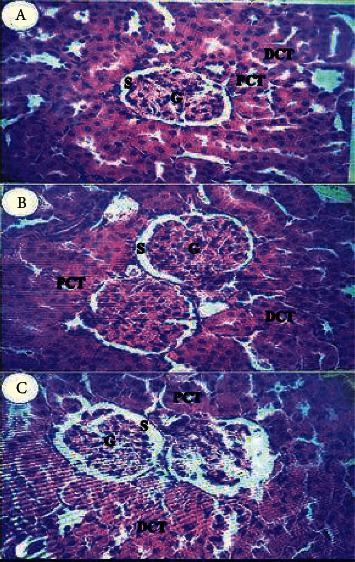
Kidneys histological sections with hematoxylin and eosin stain (200x) obtained from control rats (a), rats treated with ODSO and gentamicin (b), and rats with gentamicin (c). G, glomeruli; S, Bowman space; PCT, proximal convoluted tubules; PDT, distal convoluted tubules.

The normal renal corpuscle includes a tuft of capillaries, glomeruli, enclosed by a double-walled epithelial structure named the Bowman capsule. The urinary or Bowman space is located between the two layers of the capsule. Convoluted tubules lined with epithelium cuboid cells. Proximal convoluted tubule (PCT) profiles included especially the renal cortex with narrow and irregular light. Although, the distal convoluted tubules (DCT) possessed clear light (Figure 5(a)). In contrast, the group treated with gentamicin had renal corpuscles formed from atrophied glomeruli with expanded Bowman spaces (Figure 5(b)). Concomitant administration of ODSO and gentamicin restored the histopathological insult induced by the gentamicin. The renal histological sections of this group (Figure 5(c)) showed normal glomeruli, each containing a tuft of glomerular capillaries surrounded by visceral and parietal layers of the Bowman capsule separated by a narrow space of Bowman.

## 4. Discussion

Clinically, gentamicin is a broad-spectrum bactericidal antibiotic aminoglycoside, commonly used alone or in combination with an active drug for the control of serious and fatal infections induced by Gram-positive and Gram-negative aerosols [24]. The main side effect of gentamicin therapeutic doses is nephrotoxicity. It was documented that up to 30% of patients treated for more than 7 days with gentamicin had some symptoms of nephrotoxicity (induced proximal tubular lesions) [25–27]. Wherefore, the nephroprotective effect of ODSO was evaluated against gentamicin-induced kidney damage. The results of this study showed that daily administration of ODSO with gentamicin alleviated significantly the side effect of gentamicin on the kidney. The injection of gentamicin caused an increase in urea and creatinine levels, which are considered biomarkers of nephrotoxicity. High levels of serum creatinine and urea are indicators of kidney damage and dysfunction, and they are mainly due to the reduction of glomerular filtration [28]. The pretreatment of rats with ODSO three hours before the injection of gentamicin has prevented gentamicin-induced kidney damage by mitigating the increase in creatinine and urea levels. However, no significant changes in the plasma electrolytes, such as sodium, potassium, chloride, phosphate, and calcium, were noted in all studied groups. Gentamicin is assumed to be associated with the production of reactive oxygen species in the form of superoxide anion (O2^−^), hydrogen peroxide (H_2_O_2_), and hydroxyl radical (OH^•^) in renal cortical mitochondria, which are accompanied by an increase in lipid peroxidation [29]. Changes in the lipid composition of the membrane could be the result of lipid peroxidation initiated by free radicals with a subsequent increase in MDA, one of the products of lipid peroxidation [30]. In this study, the daily administration of gentamicin to rats caused an increase in lipid peroxidation, which is indicated by the elevation of renal MDA. Moreover, the pretreatment of the rats with ODSO three hours before the injection of rats prevented gentamicin-induced lipid peroxidation by mitigating the increase of MDA. Besides, the histopathological examination of the kidney tissue showed that gentamicin induces kidney tissue injury (by-products of lipid peroxidation); however, the rats treated with ODSO did not show any histopathological change in the kidney tissue. The effect of the oil could be related to the increase in the enzymatic antioxidant activity. Furthermore, phytochemical studies have shown that this oil is rich in antioxidant elements. As a matter of fact, this oil is characterized by a great level of unsaturated fatty acids, especially linoleic acid and *β*-sitosterol as a sterol marker and *γ*-tocopherol as vitamin E [14]. Moreover, Abdel-Naim et al. indicated that vitamin E and its antioxidant activity have a potential protective effect against gentamicin-induced nephrotoxicity [31]. These findings were supported by Tomasch et al. which demonstrated that *γ*-tocopherol rich oil intake improves plasmatic antioxidant capacity [32]. A study that concerns the chemical composition of ODSO has shown that it is also rich in phenolic compounds [33]. These compounds are well known for their antioxidants power, and they are found in many foods and beverages and involved in the prevention of several major chronic illnesses [34, 35]. Therefore, the nephroprotective effect of ODSO may be related to its free radical trapping effect generated by gentamicin and related to its antioxidant activities. The results in the present work are consistent with the results found by Bouhrim et al. They found that this oil offered a protective effect against oxidative stress caused by CCl_4_ by reducing the elevation of hepatic MDA [17]. Concerning the increase of liver enzymes by the high dose of gentamicin is similar to the findings of other studies [36]. The increase of ALP is an indication of liver damage [37]. In addition, a recently published study showed that an increase in the rate of serum albumin indicates hepatotoxicity [38]. The present study revealed that the treatment by ODSO maintained almost normal plasma albumin and ALP when compared with the control group, which is most likely due to the preservation of liver function. The results of this study are in agreement with other studies that showed the ability of alternatives natural products to prevent hepatotoxicity by drug-derived [39]. However, nonsignificant changes in plasma GGT concentration were observed in all groups studied. The variation in weight gain across the groups of treated rats was not significant. Indeed, no significant increase in weight gain was observed in rats treated only with gentamicin. This variation was slightly restored by ODSO. Besides, the right kidney weight in the gentamicin group decreased slightly compared to the control group, which could be due to edema caused by a malfunction in the reabsorption process and a reduction in renal glomerular filtration [40].

## 5. Conclusion

This study demonstrated ODSO effects elevating renal toxicity caused by gentamicin overdose. ODSO significantly improved different biochemical and histological parameters compared to the nontreated group. The study also was conducted in terms to look for new safe and effective agents to treat the liver, kidney, and other organs' dysfunctions and diseases. Although, more studies are required to explore exactly the ODSO action mechanism against gentamicin-induced physiological disturbances and histopathological alteration.

## Figures and Tables

**Table 1:** Comparison of the renal biomarkers of the animal models.

Groups	Creatinine (mg/L)	Uric acid (mg/L)	Urea (g/L)
NCG	3.16 ± 0.05	8.33 ± 2.51	0.26 ± 0.07
GCG	4.56 ± 0.35^*∗∗*^	19.33 ± 1.15^*∗∗∗*^	0.56 ± 0.05^*∗∗∗*^

The values are expressed in mean ± SEM (*n* = 3). ^*∗∗*^*P* < 0.01 and ^*∗∗∗*^*P* < 0.001, compared to normal control group (NCG).

**Table 2:** Effect of ODSO administration on bodyweight gain and right kidney weight variation.

Groups	NCG	GCG	OOG
Weight gain(g)	28.20 ± 16.81	32.47 ± 20.74	30.80 ± 02.64
Relative right kidney weight (g/100 g BW)	00.39 ± 00.30	00.31 ± 00.02	00.35 ± 00.02

**Table 3:** Effect of ODSO on plasma electrolyte levels in rats with or without gentamicin administration.

Groups	NCG	GCG	OOG
Na^+^ (mM)	138.33 ± 1.53	136.5 ± 0.70	135.33 ± 1.53
K^+^ (mM)	4.93 ± 0.75	4.35 ± 0.07	4.63 ± 0.25
Cl^−^ (mM)	105 ± 1.73	104.5 ± 0.70	101.33 ± 0.57
P (mM)	62.65 ± 13.04	74.9 ± 15.90	56.56 ± 13.35
Ca^+^ (mM)	89.75 ± 11.97	95.72 ± 4.64	91.04 ± 11.40

## Data Availability

The data used to support the findings of this study are available from the corresponding author upon request.
